# Frequency tongue coating in patients referred to Kerman Dental School and its relationship with relative factors

**DOI:** 10.1186/s12903-023-03306-2

**Published:** 2023-08-25

**Authors:** Elham Abbaszadeh, Nader Navabi, Saghar Karimi Afshar, Maryam Alsadat Hashemipour

**Affiliations:** 1 Department of Oral Medicine, School of Dentistry, Kerman University of Medical Sciences, Kerman, Iran; 2 Oral and Dental Diseases Research Center, Kerman University of Medical Sciences, Kerman, Iran; 3 Private Practice, Kerman, Iran; 4 Social Determinants On Oral Health Research Center, Kerman University of Medical Sciences, Kerman, Iran

**Keywords:** Hairy tongue, Simplified Oral Health Index, Frequency

## Abstract

**Objective:**

There has been shown a relationship between “tongue coating” and “Simplified Oral Health Index, periodontal status, modified mallampati classification (MMC) of the oropharynx and oral malodor”. The purpose of this study is to assess the frequency of tongue coating and relative factors (sex, age, smoking, systemic disease and oral health indices) among patients referred to Dental School of Kerman University.

**Methods:**

In this cross sectional study 250 patients referred to dental school of Kerman university of medical sciences were examined. The data collection form was included demographic data (gender, age, history of systemic disease and smoking) and Oral health indices such as TCI (Tongue coating index), OHI-S (Simplified Oral Hygiene Index), MGI (Modified Gingival Index), MMC and lost teeth. The analysis have been done using SPSS21, T-test, Pearson correlation coefficient and linear regression analysis (significance level was set at less than 0.05).

**Results:**

Tongue coating has been shown in 96% of patients with the mean percent of 45.83 ± 19.16%. Men had higher percent of TCI though it was not statistically significant. Smoking was the strongest determinant factor in people with higher TCI scores (*P* = 0.013). There was a positive significant correlation between OHI-S and TCI [(Pearson’s coefficient(r) = 0.134, *P* = 0.034)].

**Conclusion:**

TCI appears to be related to smoking and Simplified Oral Health Index. The evaluation of tongue coating is necessary to assess its impact on oral health status and also to motivate patients to clean their tongue as a part of their oral health care routine.

## Introduction

The tongue is an organ accessible through the oral cavity and has been used for thousands of years as an index of general health in Western and Eastern medicine [1, 2]. The tongue plays diverse roles, including taste perception, swallowing, talking, and mandibular development, changes in oral condition can lead to alterations in these roles [3].

Tongue lesions make up a major part of oral cavity lesions and extensive epidemiologic studies have been performed throughout the world concerning the prevalence of tongue lesions in different populations [4–6]. The papillary structure of the dorsal surface of the tongue forms a unique ecologic site providing a wide surface for the accumulation of oral debris and microorganisms [7]. The usual appearance of the dorsal surface is pink with or without a thin white layer [8].

The tongue coating is a white-brown visible layer located at the dorsal surface of the tongue consisting of epithelial cells, blood cells, metabolites, nutrients, and bacteria [9, 10]. It is known as the most prevalent tongue condition with different prevalence in distinct parts of the world [9]. This diversity might be due to the race, gender, and age of the investigated subjects along with differences in diagnostic criteria, methodology, and sampling method of the researchers [11].

Evaluation of the Tongue Coating Index (TCI) is a valid, simple, and reliable visual method for measuring tongue coating [12]. In order to assess the impact of oral health care and encourage the patients for tongue cleaning, it is necessary to evaluate tongue coating status. The present study aimed to investigate the frequency and rate of tongue coating among the patients referring to the Dentistry Faculty of Kerman, Iran, and to evaluate its relationship with other oral health indices.

## Methods

This descriptive cross-sectional study was conducted on 250 patients referred to department of Oral Medicine of Kerman University of Medical Sciences, from January 2019 to July 2020.

### Ethics statement

The study was approved by the ethics committee of Kerman University of Medical Sciences (IR.KMU.REC.1397.506) by the research deputy of Kerman University of Medical Sciences. Oral informed consent was obtained from all patients for examinations and participation in the study following the provision of the needed explanations. All the information on the subjects will remain confidential. The procedure of obtaining verbal informed consent was approved by an ethics committee.

### Study participants

All subjects (250 cases) attended for routine dental check up or for dental treatment in departments restorative, oral medicine, orthodontics… were included in this study. This study was part of the regular appointment of patients who referred to the faculty for treatment. Sample size calculation was completed considering the prevalence of 20% for the coated tongue [13] and using the following equation: N = Z^2^ P Q / D^2^ ➔ N ~ 250, P = 20% Q = 80% Z = 1.96 D = 0.05.

Based on a sampling approach, 250 female and male mean age 30.2 ± 9.8 years presenting for routine screening were recruited consecutively for this study.

Inclusion criteria were age over 18 years, no use of antibiotics and mouthwash in the last two weeks and no pregnancy. The oral examinations of tongue coating were performed with plain mouth mirrors under on a dental unit. A final year student who was trained by an Oral Medicine specialist measured the following indicators measured.

The following indexes:

1. TCI

Tongue coating condition was evaluated utilizing the criteria proposed by Gómez et al. and winkle et al. [14, 15]. The tongue of each patient was subjectively divided into three longitudinal and traverse parts resulting in nine equal parts. Each of the nine parts was scored based on the criteria in Table 1 and the scores were recorded in the relevant part of the Tongue Coating Record (TCR). In the case of observing several conditions in one part, the coating with the most area was regarded as the final score. The final score was calculated as a percentage utilizing the following formula:

**Table 1:** Scoring criteria for tongue coating index

Code	Scoring criteria
0	The tongue coating is not visible
1	Thin tongue coating with visible papilla
2	Very thick tongue coating with invisible papilla

$$\mathrm{TCI }= (\text{total score of 9 sections/18)}* 100$$

For the final statistical calculations, scores 1 and 2 were regarded as TCI ≤ 50% and TCI > 50%, respectively.

2- Simplified Oral Health Index (OHI-S)

The OHI-S was developed to classify and evaluate oral health for accessing a systematic method of quantifying oral health factors in population studies [16]. This index entails two parts: The simplified Debris Index (DI-S) and Simplified Calculus Index (CI-S). Each of these two indices is based on numeric determinants that demonstrate the amount of debris or calculus found on previously selected teeth.

In this index, each of the buccal and lingual surfaces of the tongue is considered half of the oral environment. In addition, only the permanent teeth that have full growth are scored. Following the selection of the six surfaces of teeth, the determined scores are recorded and calculated for DI-S and CI-S.In each individual, first, the debris and calculus scores are separately summed and divided by the number of the scored dental surfaces. Afterwards, the scores are summed to obtain the OHI-S. The total scores of 0.1–1.2, 1.3–3, and 3.1–6 were categorized as good, moderate, and weak, respectively. For statistical analysis, the scores of 1, 2, and 3 were regarded for good, moderate, and weak conditions, respectively.

3- Modified Gingival Index (MGI)

This index is applied to assess the prevalence and severity of gingivitis in a non-invasive and visual way. In order to obtain MGI, the labial, facial, and lingual surfaces of the selected teeth are examined from the gingival ridge and interdental papilla after soft drying of the regions by air current or cotton roll. The examination and scoring are performed under sufficient light and the severity of gingivitis was determined based on gingival examination. For calculating MGI in each person, the scores of papillary and marginal gingiva were summed and the outcome was divided by the number of examined units. The scores of 0.1–1, 1.1–2, and > 2.1 were representative of mild, moderate, and severe inflammation, respectively. For the statistical analysis, these conditions were scored as 1, 2, and 3, respectively [17].

4- Mallampati Classification (MMC) [18]

The MMC is a standard method for classifying pharyngeal soft tissue to predict the difficulty of intubation. In order to evaluate MMC, the patient should sit completely straight, open their mouth widely, and protrude the tongue without any voice. All patients are categorized in the four following classes:

- Class I: soft palate, fauces pillars, and uvula completely visible
- Class II: soft palate, fauces pillars, and uvula visible
- Class III: soft palate and base of uvula visible
- Class IV: soft palate not visible at all.

The inclusion criteria for the present study encompassed having all the studied teeth or substitute for evaluation by OHI-S and MGI, the eruption of all permanent teeth except the third molar, and patient consent. The exclusion criteria were the lack of all posterior teeth in each quadrant, limitation for the complete opening of the mouth, and a history of dental scaling in the previous month.

In addition to the above indexes, demographic variables such as age, sex, systemic diseases and smoking and the number of lost or extracted teeth were also recorded.

The data were analyzed by the t-test, Pearson correlation, and linear regression analysis using the SPSS software version21. *P* < 0.05 was considered significant for all tests.

Frequency distribution of the dependent categorical variables was compared by the t-test. The level of significance for all the analyses was set at a value of *P* = 0.05 at 95% Confidence Interval (CI). Multivariate logistic regression analysis was used to estimate the OR and confidence interval (CI) for the association between TCI, MMC, MGI, OHI-S and the demographic characteristics for potential confounding variables.

Stratified logistic regression analyses were performed to assess the relationship between TCI, and MMC, MGI, OHI-S.The study was approved by the ethics committee of Kerman University of Medical Sciences by the research deputy of Kerman University of Medical Sciences. A statement to confirm that all experimental protocols were approved by the research deputy of Kerman University of Medical Sciences. The informed verbal consent was obtained from the participants for examinations and participation in the study following the provision of the needed explanations by the research deputy of Kerman University of Medical Sciences. All the information on the subjects will remain confidential. The procedure of obtaining verbal informed consent was approved by Research Ethics Committees, Research Ethics Committee of Kerman University of Medical Sciences and IBR No: IR.KMU.REC.1397.506. All experiments were performed in accordance with relevant guidelines and regulations (such as the Declaration of Helsinki)

## Results

A total of 250 patients (151 women and 99 men) with the mean age of 30.2 ± 9.8 years were evaluated. Among the subjects, 28 smoked and 40 had diverse systemic diseases, among which thyroid disorder (*n* = 14), blood hypertension (*n* = 11), and diabetes (*n* = 6) were more prevalent. Almost all patients had some degrees of tongue coating (96% had tongue coating and 4% had a very thin and negligible coating Table 1.

The mean ± SD of TCI was 45.83 ± 19.16. also, minimum and maximum was reported zero and 100, respectively. The mean ± SD of OHI-S was 1.02 ± 0.68. Also, minimum and maximum was reported 0.1 and 4.1, respectively. The Table 2 show frequency, percentage, Mean & SD of TCI, OHI-S, MGI, Mallampati indexes.

**Table 2:** Demographic characteristics of the participants in this study

Variable		N	%
Sex	Female	151	60.4
	Male	99	39.6
Mean Age	30.2 ± 9.8		
Age Range	25–61		
Smoking	Positive	28	11.2
	Negative	222	88.8
Systemic Disease	Positive	38	15.2
	Negative	212	84.8
Tongue Coating	Yes	240	96
	No	10	4

The scales of tongue coating in 190 and 60 patients were recorded as 1 and 2, respectively. Also, the mean percentage of tongue coating in patients was found to be 45.83 ± 19.1. 66.8 percent participant has good OHI-S. Also, 40.4% patients had severe and moderate MGI Table 3.

**Table 3:** The frequency, percentage, Mean & SD of TCI, OHI-S, MGI, Mallampati indexes

Index		No	%	Min	Max	Mean	SD
TCI	≤ 50%	190	76	0	100	45.83	19.16
	> 50%	60	24				
OHI-S	Good	167	66.8	0. 1	4.1	1.02	0.68
	Moderate	80	32				
	Poor	3	1.2				
MGI	No	2	0.8	0	3.6	1.79	0.82
	Mild	46	18.4				
	Moderate	101	40.4				
	Severe	101	40.4				
Mallampati	Class I	66	26.4	0	3.2	2.21	0.96
	Class II	96	37.2				
	Class III	63	25.2				
	Class IV	25	11.2				
Tooth lost	0	128	51.2	0	9	1.32	1.81
	1–3	91	36.4				
	≥ 4	31	12.4				

This study show that there is no significant correlation between TCI, sex, age, systemic diseases and tooth lost (*P* = 0.18, *P* = 0.86, *P* = 0.81, *P* = 0.82). Although, smoking was observed to be significantly correlated with tongue coating (*P* = 0.01). Also, OHI-S index hasnot significant correlation with demographic variables ( sex, age, systemic diseases, smoking and tooth lost). This study show that there is no significant correlation between MGI, age, systemic disease and tooth lost (*P* = 0.54, *P* = 0.86, *P* = 0.42), Although, sex, smoking was observed to be significantly correlated with tongue coating (*P* = 0.004, *P* = 0.001) Table 3.

TCI had a positive correlation with OHI-S. A There were significant relationship between mallampati with OHI-S (*r* = 0.15 & *P* = 0.02), and mallampati and MGI (*r* = 0.46 & *P* = 0.0001). The multivariate linear regression analysis, also confirmed the results of univariable tests Table 4.

**Table 4:** The relationship between TCI, OHI-S, MGI and demographic variables

Variable		N	Mean TCI	SD TCI	**P value	N	Mean OHI-S	SD OHI-S	**P value	N	Mean MGI	SD MGI	**P value
Sex	Female	151	44.59	20.58	0.18	151	0.99	0.68	0.44	151	1.67	0.80	*0.004
	Male	99	47.74	16.71		99	1.06	0.68					
Smoking	Positive	28	54.28	19.92	0.01*	28	1.04	0.62	0.90	28	2.16	0.50	*0.001
	Negative	222	44.77	18.85		222	1.02	0.69		222	1.75	0.85	
Systemic disease	Positive	38	46.53	20.53	0.81	38	1.13	0.78	0.28	38	2.01	0.73	0.86
	Negative	212	45.71	18.96		212	1.05	0.66		212	1.76	0.84	
Age	Pearson coefficient (r) = 0.05				0.86	Pearson coefficient (r) = 0.06			0.64	Pearson coefficient (r) = 0.05			0.54
Tooth lost	Pearson coefficient (r) = 0.014				0.82	Pearson coefficient (r) = 0.015			0.85	Pearson coefficient (r) = 0.016			0.42

^*^P < 0.05 is significant, **Independent T test

This analysis demonstrated that smoking and OHI-S had the highest effect on tongue coating (*P* = 0.016, *P* = 0.021, respectively). Therefore, smoking had a 9.95% increase in TCI results. In addition, we found a 4.94% elevation in people with higher OHI-S Tables 5 and 6.

**Table 5:** The relationship between the indexes and TIC

Index	Mean	Std. Deviation	Pearson coefficient (r)	*P* value
TCI	45.8346	19.16590		
OHI-S	1.0246	.68135	TCI and OHIS = 0.134^a^	0.034
MGI	1.7952	.82516	TCI and MGI = 0.048	0.45
Mallampati	2.212	.9606	TCI and Mallampati = -0.06	0.38

^a^Correlation is significant at the 0.05 level (2-tailed)

**Table 6:** Prediction of the TCI score in the linear regression model

Dependent variable: TCI	Unstandardized Coefficients		Standardized Coefficients	t	Sig
	B	Std. Error	Beta		
Sex	1.618	2.684	0.041	0.603	0.547
Age	0.106	0.145	0.052	0.729	0.467
Smoking	9.955	4.109	0.164	2.423	0.016^a^
Systemic disease	0.248	3.404	0.005	0.073	0.942
OHI-S	4.947	2.131	0.176	2.322	0.021^a^
MGI	-1.149	1.687	-0.049	-0.681	0.497
Mallampati	-2.081	1.285	-0.104	-1.620	0.107
Tooth lost	-0.635	0.756	-0.060	-0.839	0.402

^a^Significant correlation

## Discussion

The present study was conducted to evaluate the frequency and rate of coated tongue and its relationship with other oral indices in 250 patients referring to the Dentistry Faculty of Kerman. Variable degrees of the coated tongue was observed in 96% of the subjects and only 4% of the patients lacked coated tongue. Van Tornout et al. reported a prevalence of 93% for diverse levels of the coated tongue [19].

In most of the studies on the coated tongue, coating of over 50% of the tongue known as the coated tongue is introduced as one of the most prevalent oral conditions [20]. Patil et al. reported a coated tongue as the most common lesion among tongue lesions (28%) [21]. Avcu and Kanli considered coated tongue with a prevalence of 23% as the second prevalent tongue lesion after hairy tongue [11]. Moreover, Omor et al. introduced a coated tongue with 21.8% prevalence as the most prevalent oral lesion [22]. Differences in the prevalence of coated tongue in different studies may be due to differences in the study population, tools and index evaluation.

In the current investigation, 76% and 24% of the patients had a coated tongue of under 50% and over 50%, respectively. Furthermore, similar to the report of Van Tornout et al., the coating was more frequent in the middle and caudal 1/3 of the tongue [19]. We did not observe a significant difference between age groups in terms of the severity of the coated tongue, which is in line with the results of Van Tornout et al. [19]. However, Seerangaiyan et al. and Kanli and Avcu revealed a significant relationship between coated tongue and older age [9, 11]. This difference can be due to the fact that in the study Seerangaiyan et al. and Kanli and Avcu [9, 11] examined older people and language coverage increases with age.

This study show that the prevalence coated tongue is higher among men and the mean of the coated tongue index in men was slightly higher than women. Although, that there is no significant correlation between TCI, sex, age, systemic diseases and tooth lost.

Several studies evaluated the relationship of tongue lesions with gender. Patil et al. in India and Matlabnejad et al. in an epidemiologic study on 1901 Iranian patients concerning the prevalence and intensity of tongue lesions reported a higher frequency in men than women [6, 21]. In addition, in the two investigations completed by Darwazeh et al. and Omor et al. on different populations of Jordanians, the prevalence and intensity of coated tongue were higher among men. Compared to women [23, 24].

Kanli and Avcu did not find a significant difference between the two genders in this regard [11]. The difference in various studies can be attributed to the smaller sample size or the difference in the evaluation method. Also, some risk factors that may increase coated tongue include: improper oral hygiene, antibiotics, alcohol, smoking, tobacco products, illegal drugs, hypothyroidism, diabetes, syphilis, weakened immune system, trauma to mouth, dehydration and xerostomia [11].

In the current study, no significant relationship was revealed between systemic diseases and the evaluated oral indices. This could be due to the low number of patients with systemic diseases and the dispersion of diseases.

A strong relationship was noted between coated tongue and diabetes in a study on the patients of the Dentistry Department of Jordanian Royal Medical Services [24]. Patil et al. reported anemia, blood hypertension, and diabetes as the most common systemic conditions among patients with the coated tongue. However, no significant relationship was observed between these diseases and the coated tongue [21] that is similar to our study. Other studies mentioned the coated tongue to have a relationship with membranous nephropathy and gastrointestinal disorders, such as maldigestion and gastritis [1, 25, 26].

In present study, that smoking had a significant relationship with coated tongue, which is consistent with the findings of Darwazeh et al., Van Tornout et al., Avcu et al., Omor et al., and Matlabnejad et al. [6, 11, 19, 23, 24].

Also our study showed that coated tongue to have a significant relationship with Simplified Oral Health Index. Van Tornout et al. (2013) reported a significant correlation between the coated tongue and Simplified Oral Health Index [19]. Moreover, Matlabnejad et al. and Avcu et al. demonstrated a strong relationship between these two factors [6, 11]. Different studies showed that the microorganisms that cover the tongue can lead to the formation of dental plaque [27, 28]. Furthermore, a coated tongue has been noted to have a relationship with some factors, including the clinical parameters of oral hygiene, nutrition, the number of gingiva cleaning times, smoking, drinking tea and coffee, and periodontal tissue condition [19].

Goud et al. revealed a relationship between Mallampati index and other oral indices, such as tongue coat, Simplified Oral Health Index., and periodontal condition. They noted that increased Mallampati score resulted in higher scores of the coated tongue, gingival index, and Simplified Oral Health Index. in the patients, and the highest scores of OHI-S and GI were observed in the subjects with Mallampati class IV. These authors reported a significant relationship between TCI and MMC only in Mallampati classes I, II, and III [29]. However, in present study, the relationship between the Mallampati score and the coated tongue was not statistically significant. while the Mallampati index had a significant relationship with OHI-S and MGI. This difference can be due to the study population and assessment methods.

Moreover, the coated tongue was reported to be correlated with halitosis in patients without periodontal disease. No relationship was mentioned between the coated tongue and the periodontal condition of the patients [30].

Van Tornout et al. revealed the condition of periodontal tissues and gingiva as one of the effective factors with a positive correlation with coated tongue [19]. We did not find any relationship between the gingival condition and coated tongue, which might be due to the different methods of MGI evaluation. In addition, no remarkable correlation was noted between the coated tongue and the number of lost teeth. Disagreements in the results of studies can be related to differences in measurement and diagnostic methods, population and sample size.

However, this research has its limitations, which include: In this study, the method used for measurement coated tongue is visual evaluation, while microbiological assessment of the coated tongue is more accurate. Furthermore, examining all teeth for evaluating OHI-S and MGI provides more precise criteria for the oral and dental hygiene status of the patients.

## Conclusion

There is no significant correlation between TCI, sex, age, systemic diseases and tooth loss. This study show that smoking and OHI-S had the highest effect on tongue coating. The smoking had a 9.95% increase in TCI results. Also, TCI had a positive correlation with OHI-S.

Investigations with larger sample size and evaluation of coated tongue in groups of patients with systemic diseases are recommended for a more accurate assessment.

## Limitations

The tongue coverage index (TCI) and other oral health indicators rely on subjective assessments and visual observations. The lack of objective measurements or diagnostic criteria in this study may cause changes in the results. However, to reduce this case, all examinations were performed by one person to reduce this defect as much as possible.

## Data Availability

The datasets used and/or analysed during the current study available from the corresponding author on reasonable request.
